# Socioeconomic differences in childhood length/height trajectories in a middle-income country: a cohort study

**DOI:** 10.1186/1471-2458-14-932

**Published:** 2014-09-08

**Authors:** Rita Patel, Kate Tilling, Debbie A Lawlor, Laura D Howe, Natalia Bogdanovich, Lidia Matush, Emily Nicoli, Michael S Kramer, Richard M Martin

**Affiliations:** School of Social and Community Medicine, University of Bristol, Canynge Hall, 39 Whatley Road, Bristol, BS8 2PS UK; MRC Integrative Epidemiology Unit at the University of Bristol, Bristol, UK; The National Research and Applied Medicine Mother and Child Centre, Minsk, Belarus; Department of Pediatrics, Biostatistics and Occupational Health, McGill University Faculty of Medicine, Montreal, Canada; Department of Epidemiology, Biostatistics and Occupational Health, McGill University Faculty of Medicine, Montreal, Canada; National Institute for Health Research, Bristol Biomedical Research Unit in Nutrition, Bristol, UK

**Keywords:** Length, Height, Growth trajectory, Socioeconomic factors, Belarus

## Abstract

**Background:**

Socioeconomic disadvantage is associated with shorter adult stature. Few studies have examined socioeconomic differences in stature from birth to childhood and the mechanisms involved, particularly in middle-income former Soviet settings.

**Methods:**

The sample included 12,463 Belarusian children (73% of the original cohort) born in 1996–1997, with up to 14 stature measurements from birth to 7 years. Linear spline multi-level models with 3 knots at 3, 12 and 34 months were used to analyse birth length and growth velocity during four age-periods by parental educational achievement (up to secondary school, advanced secondary/partial university, completed university) and occupation (manual, non-manual).

**Results:**

Girls born to the most (versus least) educated mothers were 0.43 cm (95% confidence interval (CI): 0.28, 0.58) longer at birth; for boys, the corresponding difference was 0.30 cm (95% CI: 0.15, 0.46). Similarly, children of the most educated mothers grew faster from birth-3 months and 12–34 months (p-values for trend ≤0.08), such that, by age 7 years, girls with the most (versus least) educated mothers were 1.92 cm (95% CI: 1.47, 2.36) taller; after controlling for urban/rural and East/West area of residence, this difference remained at 1.86 cm (95% CI: 1.42, 2.31), but after additionally controlling for mid-parental height, attenuated to 1.10 cm (95% CI: 0.69, 1.52). Among boys, these differences were 1.95 cm (95% CI: 1.53, 2.37), 1.89 cm (95% CI: 1.47, 2.31) and 1.16 cm (95% CI: 0.77, 1.55), respectively. Additionally controlling for breastfeeding, maternal smoking and older siblings did not substantively alter these findings. There was no evidence that the association of maternal educational attainment with growth differed in girls compared to boys (p for interaction = 0.45). Results were similar for those born to the most (versus least) educated fathers, or who had a parent with a non-manual (versus manual) occupation.

**Conclusions:**

In Belarus, a middle-income former Soviet country, socioeconomic differences in offspring growth commence in the pre-natal period and generate up to approximately 2 cm difference in height at age 7 years. These associations are partly explained by genetic or other factors influencing parental stature.

**Trial Registration:**

Current Controlled Trials: NCT01352247 assigned 9 Sept 2005; ClinicalTrials.gov. Identifier: NCT01561612 received 20 Mar 2012.

**Electronic supplementary material:**

The online version of this article (doi:10.1186/1471-2458-14-932) contains supplementary material, which is available to authorized users.

## Background

Adult attained height reflects fetal, infant and childhood growth and hence the influence of in utero and postpartum environment and genes [1, 2]. Height is an important marker of health, because shorter people have higher cardiovascular disease risk [3, 4] and higher overall mortality [5], albeit a lower risk of developing some cancers [4–6]. Socioeconomic differences in length and/or height (abbreviated to length/height) have been demonstrated at all ages and in various settings [7–14], with socioeconomic disadvantage associated with shorter stature.

In high-income populations, some cross-sectional studies have found that socioeconomic differences in height have narrowed over the generations [15], whilst others report their persistence [16]. Socioeconomic height differences have also recently been reported in contemporary low- and middle-income populations [17, 18]. Better living conditions in high-income countries may lead to improvements in childhood health, environment and social conditions [15] and may reduce adverse environmental effects, allowing genetic potential to be reached and a reduction in socioeconomic differences. Factors such as improved infant-feeding [19], reductions in maternal smoking [20, 21] and overcrowding [19] or other environmental features may affect children’s stature. Such factors are important to examine in relation to growth because they are potentially modifiable and their distribution may change across socioeconomic groups over time. In contrast, parental height reflects both environmental and genetic influences [22] on growth but is not immediately modifiable.

Few studies have examined the development of socioeconomic differences in length/height from birth through infancy to childhood using repeated measures of stature; of those that have, most have been set in high-income countries [23–26], with fewer in low- or middle-income countries, such as Brazil [18, 27] or former Soviet countries, such as Russia [28]. It is important to understand not only the magnitude and timing of the emergence of socioeconomic differences in length/height (as these may clarify important growth periods when public health interventions aimed at reducing these differences, and hence inequalities in later health outcomes, might be targeted), but also the mechanisms involved (which may suggest modifiable factors suitable for intervention). By examining socioeconomic differences in childhood growth and the factors that affect growth in a middle-income former Soviet country, we aimed to understand whether the findings are specific to this context or share similarities with those in other settings.

Belarus is a middle-income [29], former Soviet republic, with features in common with high-income countries such as high adult literacy and low child mortality rates but, in contrast to many high-income countries, low income inequality [30] and high adult mortality rates, particularly from cardiovascular disease [31]. We have previously reported that Belarusian children of the most educated parents or non-manual households were taller at age 6.5 years than those of the least educated parents or manual households [17]. Here, we extend our earlier analysis to model individual length/height trajectories from birth to 7 years by maternal and paternal educational achievement, and parental occupation, in over 12,000 children. We examine when socioeconomic differences in length/height emerge, whether they change with age, and the effect of controlling for prolonged and exclusive breastfeeding, maternal smoking, the number of older siblings and mid-parental height to shed light on potential mechanisms generating socioeconomic differences in birth size and postnatal stature.

## Methods

### Study design and participants

The study cohort is based on 17,046 children and their mothers who were originally recruited during their postpartum hospital stay into the Promotion of Breastfeeding Intervention Trial (PROBIT) [32] in Belarus in 1996–1997. Briefly, 31 maternity hospitals and one each of their affiliated polyclinics (outpatient clinics for routine health care) were randomly assigned, within strata of urban/rural and East/West geographical location, to participate in a breastfeeding promotion intervention or to continue with prevailing practices. Inclusion criteria specified that the mothers were healthy and initiated breastfeeding, and that the infants at birth were full-term (≥37 weeks gestation) healthy singletons, weighed at least 2,500 g at birth and had a 5-minute Apgar score ≥5. Study staff estimated that only 1-2% of eligible women declined participation in the trial [33].

### Follow-up from birth to 12 months of age

During the postpartum stay, the number of other children living together in the household, parental education and occupation and maternal smoking during pregnancy were reported by the mother, and infant birth length was abstracted from medical records [34]. The number of other children in the household was categorized as 0, 1 and 2 or more and used as a proxy for older siblings and overcrowding. Educational status was recoded from seven to three categories: initial or incomplete or common secondary; advanced secondary or partial university; and completed university, with ‘unknown’ coded as missing. Each parent’s occupation was classified as manual or non-manual and the highest household occupation determined [17]. Mother’s current smoking status and infant length was recorded by study paediatricians on scheduled study visits when the infant was aged 1, 2, 3, 6, 9 and 12 months; home visits were made when polyclinic visits were missed [34]. As differences in length/height gain were not major hypotheses of PROBIT, no attempt was made to standardise the measurement of length in infancy.

### Follow-up between 12 months to 6.5 years

In 2002–2005, at the age of 6.5 years (interquartile range (IQR): 6.5-6.7, range: 5.6-8.5 years), 13,889 children (81% of those randomized) were examined at a research visit by one of 38 polyclinic paediatricians who received special training in anthropometry. Standing height was measured twice by a standardised protocol, using a stadiometre with a movable headboard (Medtechnika, Pinsk, Belarus) and the average of the two measurements used [35]. An audit of the height measures for 190 children at 5.3-32.6 months (mean 17.7 months) after the initial 6.5-year research examination, showed a test-retest correlation of 0.84. The study paediatricians also retrospectively abstracted height data from the medical records of each child, as height was routinely measured and documented at check-ups between 12 months and 6.5 years. Of 12,463 children with complete data, 10,389 children had 39,205 measurements (median, 5; IQR, 4–5; range, 1–6) abstracted from medical records between the 12-month and 6.5-year examinations. The observed length/height measurements were converted to age- and sex-adjusted z-scores according to World Health Organization (WHO) Child Growth Standards 2006, using the STATA command *zanthro* [36].

Parental height was obtained by interview with the parent who accompanied the child at the 6.5-year follow-up visit. Any parental height that was implausible (greater than +/- 4 standard deviations (SD) from the parent-specific mean) was recoded to “missing”. Mid-parental height was calculated as the average of the two parents’ heights. Maternal current smoking status was also recorded at the 6.5-year follow-up. All occasions when maternal smoking was recorded (i.e. during pregnancy, at clinic visits during the first-year of follow-up and at 6.5 year follow-up) were recoded to yes/no from the number of cigarettes smoked per day and combined to create a longitudinal measure of ‘maternal smoking’ categorised as unknown, ever or never.

### Ethics

PROBIT was approved by the Belarusian Ministry of Health and received ethical approval from the institutional review board of the Montreal Children’s Hospital for both the original PROBIT trial and the 6.5-year follow-up. The participating parent/guardian signed consent forms in Russian at each phase.

### Statistical methods

#### Summarizing individual length/height gain trajectories

We chose the maximum age of 7 years (84 months) as our upper age cut-off, as this was the oldest age with sufficient data to describe the growth trajectory. Length/height growth velocity was predicted for each child by a linear spline multilevel model with 3 knots at 3, 12 and 34 months, the knots were chosen that best fitted the data. Full details of the model selection process have been published elsewhere [37, 38]. The knot points generated four splines, estimating different length/height growth rates between 0–3 months, 3–12 months, 12–34 months and 34–84 months; designated as ‘early infancy’, ‘late infancy’ , ‘early childhood’ and ‘late childhood’ , respectively. The model assumes piecewise linear growth and is therefore a simplification of the underlying growth process. Although a linear spline model is an approximation of the true growth function; its coefficients are easily interpretable and have been shown to produce good model fit in this and several other cohorts [18, 23, 37–40]. Furthermore, analyses in four other cohorts in different settings have resulted in similar knot points, providing some face validity that the growth periods identified represent meaningful distinct periods of growth that are similar across children from different ethnic and socioeconomic backgrounds [18, 23, 37–40]. Individual- and occasion-level residuals were approximately normally distributed. Our approach assumes that residuals are uncorrelated and that any missing outcome data are missing at random. However, even if the autocorrelation assumption is violated, the fixed effects, which we report, are likely to be unbiased [41]. The advantage of this method of growth modelling is that the model allows for the change in scale and variance of length/height over time; each child is included in the analysis; all measurements are included, regardless of when the measurements were taken (however irregularly spaced); and the actual age at measurement is taken into account. The multilevel model was fitted for three levels: i) the measurement occasion; ii) the individual child; and iii) hospital/polyclinic site where the child was examined.

The growth trajectories were modelled for all children with at least two measures of length/height (N = 16,861) and then modelled restricted to those with complete data on all covariables (N = 12,463). As findings did not differ substantively between these two datasets, we present results for those with complete data only.

#### Crude and multiple analysis of growth

The coefficients from the models were used to predict length/height measurements at various ages by socioeconomic position and to calculate the absolute difference between extreme categories of socioeconomic position (See Additional file 1: supplementary information). To allow comparison of length/height differences in Belarus with an international growth standard, we report the absolute difference in terms of standard deviations of the WHO Child Growth Standards [42], calculated by dividing the absolute difference (cm) by the standard deviation (cm) of the WHO reference length/height measurement of the same age and sex. As length/height variance widens with age, reporting findings as standard deviations also allows a comparison of the differences on the same relative scale.

Three multivariable multilevel models were fitted for growth trajectories by categories of socioeconomic position. Model 1 adjusted for urban/rural and East/West location as these geographical variables were used to stratify the original randomization and may confound the associations examined. Model 2 additionally adjusted for mid-parental height, a proxy for genetic and environmental factors that influenced the parents’ own growth [14, 19–21, 43–45], while model 3 also adjusted for trial intervention arm (as an unbiased measure of prolonged and exclusive breastfeeding), maternal smoking, and number of older siblings (because previous studies have shown these factors [19–21, 46–48] to be associated with both statural growth and socioeconomic position [49, 50]). These latter factors could be conceptualized as mediators, as poorer socioeconomic circumstances are associated with reduced breastfeeding [51], increased maternal smoking both pre- and postnatally [49, 50] and larger family size [19]. In addition, breastfeeding is associated with rapid linear growth in the first 3 months of life and less rapid growth to 12 months [48]. Maternal smoking, particularly during pregnancy, [20] is associated with shorter offspring stature. Ovecrowding or having older siblings may also increase the frequency of childhood infection [19], lead to a sharing of resources (both material and psychosocial [47]) and affect the childhood environment and conditions, all of which may impact growth adversely.

We included trial randomisation arm as the best assessment of the mediating effect of breastfeeding on child length/height [48]. The proportion of women who were exclusively breastfeeding was seven-fold higher in the intervention arm at 3 months compared to the control arm (43.3 vs. 6.4%) and more than 12-fold higher at 6 months (7.9 vs. 0.6%) [34]. To separate the effects of each parent’s height on socioeconomic differences in growth, we also replaced mid-parental height in the final model with either maternal or paternal height.

We found strong statistical evidence of a difference in the growth trajectory between girls and boys (p < 0.001), hence we present results for girls and boys separately. However, there was no evidence that the association between maternal education and the length/height growth trajectory differed in girls compared to boys (p for sex-interaction = 0.45). We tested the assumption of a linear relationship between categories of the socioeconomic exposures and length/height trajectory using likelihood ratio tests based on the fully adjusted models. As evidence against the assumption of linearity was weak (p = 0.13 for girls and p = 0.09 for boys) we present linear models. Because of the large quantity of data, and patterns that were similar across socioeconomic groups, we present the results for maternal education in detail and provide the results for paternal education and highest household occupation as supplementary Web Tables. Analyses were conducted in STATA version 13.1 (Stata Corporation, Texas) and using the *runmlwin* command [52] in MLwiN (Version 2.30) [53].

## Results

Table 1 compares the 16,861 children with at least two measures of length/height to those 12,463 with complete data and reveals little difference in the proportions of each covariable between the two data sets. Those with missing data were more likely among children of mothers with no more than a secondary school education compared to those of mothers educated to advanced secondary, partial university or completed university. Those with incomplete data were slightly shorter and lighter at birth and were more likely to come from urban or Eastern areas, be in the breastfeeding promotion (intervention) arm of the trial and be first born.

**Table 1:** **Characteristics of 16,861 children with at least two length/height measurements and 12,463 with complete data on all covariables**

	Girls										Boys									
	All available girls			Those with complete data			Those with incomplete data			P for diff ^1^	All available boys			Those with complete data			Those with incomplete data			P for diff ^1^
N	8,139			6,010			2,129				8,722			6,453			2,269			
	n	Mean	SD ^2^	n	Mean	SD	n	Mean	SD		n	Mean	SD	n	Mean	SD	n	Mean	SD	
Birth length, cm	8,139	51.6	2.0	6,010	51.7	2.1	2,129	51.4	2.0	<0.001	8,722	52.3	2.2	6,453	52.3	2.2	2,269	52.1	2.2	<0.001
Birth weight, kg	8,139	3.4	0.4	6,010	3.4	0.4	2,129	3.3	0.4	<0.001	8,722	3.5	0.4	6,453	3.5	0.4	2,269	3.5	0.4	0.004
Mid-parental height^3^	6,010	170.2	4.9	6,010	170.2	4.9	0	-	-	-	6,453	170.2	4.9	6,453	170.2	4.9	0	-	-	-
	**n**	**%**		**n**	**%**		**n**	**%**			**n**	**%**		**n**	**%**		**n**	**%**		
Maternal education																				
Initial/incomplete/common secondary	2,943	36		2,075	35		868	41		<0.001	3,143	36		2,252	35		891	39		0.001
Advanced secondary/partial university	4,085	50		3,099	52		986	46			4,395	50		3,321	51		1,074	47		
Completed university	1,111	14		836	14		275	13			1,184	14		880	14		304	13		
Urban residence (versus rural)	4,715	58		3,287	55		1,428	67		<0.001	5,172	59		3,567	55		1,605	71		<0.001
Residence in West Belarus (versus East)	4,135	51		3,148	52		987	46		<0.001	4,503	52		3,483	54		1,020	45		<0.001
In breastfeeding promotion arm	4,236	52		3,023	50		1,213	57		<0.001	4,517	52		3,219	50		1,298	57		<0.001
Maternal smoking																				
Unknown	1,682	21		239	4		1,443	68		<0.001	1,807	21		277	4		1,530	67		<0.001
Ever	1,017	13		750	12		267	13			1,089	12		788	12		301	13		
Never	5,440	67		5,021	84		419	20			5,826	67		5,388	84		438	19		
Older siblings^4^																				
None	4,694	58		3,307	55		1,387	65		<0.001	5,059	58		3,582	56		1,477	65		<0.001
1	2,761	34		2,187	36		574	27			2,912	33		2,311	36		601	27		
2 or more	684	8		516	9		168	8			750	9		560	9		190	8		

^1^p for difference between those with complete data to those with incomplete data, for those with incomplete data any data available is compared (ttest and chi squared test).

^2^SD = standard deviation.

^3^2,129 girls and 2,269 boys had missing information on mid-parental height recorded when the child was aged 6.5 years.

^4^1 boy had missing information on older siblings recorded at child’s birth.

**Figure 1:**
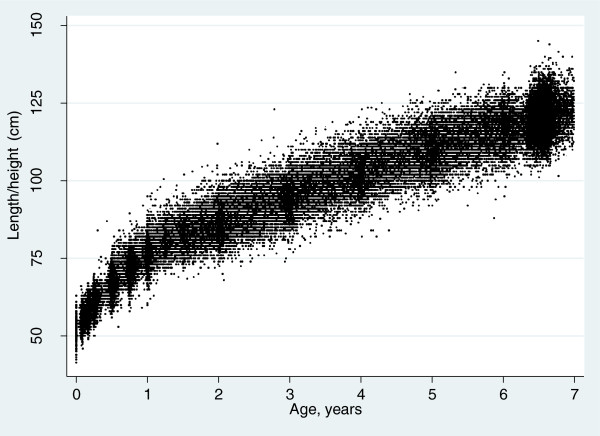
**Length/height measurements with age.**

**Figure 2:**
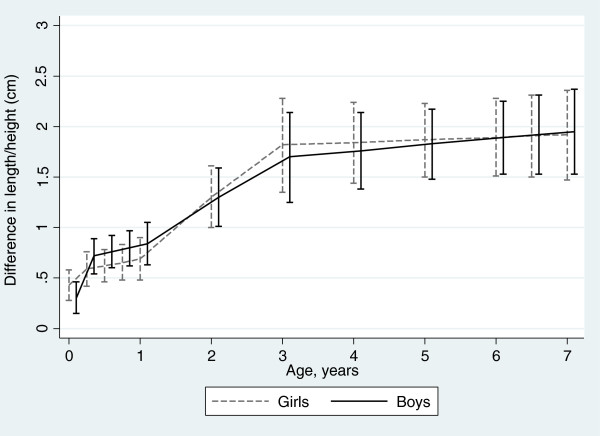
**Absolute difference in length/height, cm (95% confidence interval) between highest and lowest categories of maternal education.**

Our study sample consisted of 12,463 individuals (73% of the original cohort) with complete data on all covariables, with 133,934 measurements (median, 12; IQR, 10–13; range, 3–14). Figure 1 shows the scatterplot of length/height measurements by child’s age; each black dot represents a single data point. Additional file 1: Table S1 shows the observed lengths/heights in cm and in WHO length/height-for-age z-score, by category of maternal education. For girls aged between 6.8 and 7 years, those of the least educated mothers had a mean z-score of 0.22 (SD: 0.94), while for girls of the most educated mothers, the mean z-score was 0.61 (SD: 0.93); for boys the respective z-scores were 0.08 (SD: 0.90) and 0.64 (SD: 1.00). Predicted offspring lengths/heights per category of maternal education and age are shown in Table 2. A linear relationship across the three maternal education categories is apparent for birth length and length/height at all ages, for both girls and boys. The absolute difference in birth length between infants in the highest versus the lowest category of maternal education was 0.43 cm (95% CI: 0.28, 0.58) for girls and 0.30 cm (95% CI: 0.15, 0.46) for boys. Absolute differences in length/height between the highest versus the lowest category of maternal education increased with age, such that by age 7 years, the absolute difference was 1.92 cm (95% CI: 1.47, 2.36) among girls and 1.95 cm (95% CI: 1.53, 2.37) among boys. Table 2 also shows the absolute differences in length/height by maternal education in terms of standard deviations of WHO length/height-for-age reference; at age 7 years, this is equivalent to 0.35SD (95% CI: 0.27, 0.43) among girls and 0.37 SD (95% CI: 0.29, 0.45) among boys. The absolute SD differences in length/height between the highest versus the lowest education categories showed some widening with age. Figure 2 illustrates the absolute difference in length/height in cm, with 95% confidence interval, between the highest and lowest categories of maternal education for girls and boys.

**Table 2:** **Crude model of predicted length/height by category of maternal education, N = 12,463**

	Mean predicted length/height by category of maternal education, cm						Completed university compared to initial/incomplete/common secondary	
	(95% confidence interval, CI)							
Age	Initial, incomplete or common secondary		Advanced secondary or partial university		Completed university		Absolute difference in length or height, cm (95% CI)	Difference in length or height, in WHO [42] standard deviations
**Girls,**								
**N = 6,010**	**2,075**		**3,099**		**836**			
Birth	51.30	(51.12, 51.48)	51.52	(51.35, 51.68)	51.73	(51.54, 51.92)	0.43 (0.28, 0.58)	0.23 (0.15, 0.31)
3 months	60.22	(60.04, 60.40)	60.51	(60.34, 60.68)	60.81	(60.61, 61.00)	0.59 (0.42, 0.76)	0.28 (0.20, 0.36)
6 months	65.42	(65.24, 65.59)	65.73	(65.56, 65.89)	66.04	(65.84, 66.23)	0.62 (0.46, 0.78)	0.27 (0.20, 0.34)
9 months	70.61	(70.43, 70.79)	70.94	(70.77, 71.11)	71.27	(71.07, 71.46)	0.65 (0.48, 0.83)	0.27 (0.20, 0.34)
1 year	75.81	(75.61, 76.00)	76.15	(75.98, 76.33)	76.50	(76.28, 76.71)	0.69 (0.48, 0.90)	0.27 (0.19, 0.35)
2 years	85.41	(85.19, 85.64)	86.06	(85.87, 86.25)	86.71	(86.45, 86.98)	1.30 (1.00, 1.61)	0.40 (0.31, 0.50)
3 years	94.56	(94.27, 94.86)	95.47	(95.24, 95.70)	96.38	(96.02, 96.73)	1.82 (1.35, 2.28)	0.48 (0.35, 0.60)
4 years	101.61	(101.35, 101.88)	102.53	(102.32, 102.74)	103.45	(103.14, 103.77)	1.84 (1.44, 2.24)	0.43 (0.33, 0.52)
5 years	108.66	(108.41, 108.91)	109.60	(109.39, 109.80)	110.53	(110.23, 110.83)	1.87 (1.50, 2.23)	0.39 (0.32, 0.47)
6 years	115.71	(115.46, 115.97)	116.66	(116.45, 116.87)	117.61	(117.30, 117.91)	1.89 (1.51, 2.28)	0.37 (0.29, 0.44)
6.5 years	119.24	(118.97, 119.50)	120.19	(119.98, 120.40)	121.14	(120.82, 121.47)	1.91 (1.50, 2.31)	0.36 (0.28, 0.44)
7 years	122.76	(122.48, 123.04)	123.72	(123.50, 123.94)	124.68	(124.34, 125.03)	1.92 (1.47, 2.36)	0.35 (0.27, 0.43)
**Boys,**								
**N = 6,453**	**2,252**		**3,321**		**880**			
Birth	52.00	(51.83, 52.17)	52.15	(51.99, 52.31)	52.30	(52.12, 52.48)	0.30 (0.15, 0.46)	0.16 (0.08, 0.24)
3 months	61.41	(61.24, 61.59)	61.77	(61.61, 61.93)	62.13	(61.94, 62.32)	0.72 (0.54, 0.89)	0.35 (0.26, 0.44)
6 months	66.58	(66.41, 66.75)	66.96	(66.80, 67.12)	67.34	(67.15, 67.52)	0.76 (0.60, 0.92)	0.35 (0.28, 0.43)
9 months	71.75	(71.58, 71.92)	72.15	(71.99, 72.31)	72.54	(72.35, 72.73)	0.80 (0.62, 0.97)	0.35 (0.28, 0.43)
1 year	76.92	(76.73, 77.10)	77.34	(77.17, 77.50)	77.75	(77.55, 77.96)	0.84 (0.63, 1.05)	0.35 (0.26, 0.44)
2 years	86.41	(86.20, 86.62)	87.06	(86.88, 87.24)	87.71	(87.46, 87.96)	1.30 (1.01, 1.59)	0.43 (0.33, 0.52)
3 years	95.44	(95.17, 95.72)	96.29	(96.08, 96.50)	97.14	(96.80, 97.48)	1.70 (1.25, 2.14)	0.46 (0.34, 0.58)
4 years	102.33	(102.09, 102.58)	103.21	(103.02, 103.41)	104.09	(103.80, 104.39)	1.76 (1.38, 2.14)	0.42 (0.33, 0.51)
5 years	109.22	(108.99, 109.46)	110.14	(109.95, 110.33)	111.05	(110.77, 111.33)	1.83 (1.48, 2.17)	0.39 (0.32, 0.47)
6 years	116.11	(115.88, 116.35)	117.06	(116.86, 117.25)	118.00	(117.71, 118.29)	1.89 (1.53, 2.25)	0.38 (0.31, 0.46)
6.5 years	119.56	(119.31, 119.81)	120.52	(120.32, 120.72)	121.48	(121.18, 121.79)	1.92 (1.53, 2.31)	0.38 (0.30, 0.45)
7 years	123.01	(122.74, 123.27)	123.98	(123.77, 124.19)	124.96	(124.63, 125.28)	1.95 (1.53, 2.37)	0.37 (0.29, 0.45)

Table 3 shows the change in length/height rate per category of mother’s education during early infancy (birth to 3 months) was 0.31 cm/year (95% CI: -0.03, 0.65; p for trend = 0.08) for girls and 0.83 cm/year (95% CI: 0.48, 1.17; p for trend < 0.001) for boys. However, in late infancy (3–12 months) and late childhood (34–84 months) we found only weak evidence that growth rates varied between categories of maternal education for both girls and boys (all p-values for trend ≥0.25). In early childhood (12–34 months) we found strong evidence that growth rates were higher per category of mother’s education: for girls by 0.31 cm/year (95% CI: 0.18, 0.43; p for trend < 0.001); and for boys by 0.23 cm/year (95% CI: 0.11, 0.35; p for trend <0.001).

**Table 3:** **Crude & multiple analysis of mean growth rate per category of maternal education, N = 12,463**

	Per category of mother’s education change in length/height rate											
	Unadjusted model			Model 1			Model 2			Model 3		
	^1^Coef	95% CI	P for trend	Coef	95% CI	P for trend	Coef	95% CI	P for trend	Coef	95% CI	P for trend
**Girls N = 6,010**												
Birth length, cm	0.22	(0.14, 0.29)	<0.001	0.24	(0.17, 0.32)	<0.001	0.20	(0.12, 0.27)	<0.001	0.17	(0.10, 0.25)	<0.001
Growth, cm/year:												
0-3 months	0.31	(-0.03, 0.65)	0.08	0.11	(-0.23, 0.44)	0.53	-0.03	(-0.37, 0.31)	0.85	-0.04	(-0.38, 0.30)	0.81
>3-12 months	0.07	(-0.07, 0.20)	0.33	0.06	(-0.08, 0.20)	0.39	0.01	(-0.13, 0.14)	0.94	0.02	(-0.12, 0.16)	0.79
>12-34 months	0.31	(0.18, 0.43)	<0.001	0.28	(0.15, 0.41)	<0.001	0.22	(0.10, 0.35)	0.001	0.25	(0.12, 0.38)	<0.001
>34-84 months	0.01	(-0.05, 0.08)	0.71	0.03	(-0.04, 0.09)	0.45	-0.01	(-0.08, 0.05)	0.72	-0.02	(-0.09, 0.05)	0.56
**Boys N = 6,453**												
Birth length, cm	0.15	(0.07, 0.23)	<0.001	0.19	(0.11, 0.27)	<0.001	0.13	(0.06, 0.21)	0.001	0.11	(0.03 , 0.18)	0.005
Growth, cm/year:												
0-3 months	0.83	(0.48, 1.17)	<0.001	0.50	(0.16, 0.84)	0.004	0.37	(0.03, 0.70)	0.03	0.38	(0.04 , 0.72)	0.03
>3-12 months	0.08	(-0.06, 0.22)	0.25	0.08	(-0.06, 0.22)	0.27	0.04	(-0.10, 0.18)	0.57	0.04	(-0.11 , 0.18)	0.62
>12-34 months	0.23	(0.11, 0.35)	<0.001	0.21	(0.09, 0.33)	0.001	0.16	(0.04, 0.28)	0.01	0.17	(0.04 , 0.29)	0.008
>34-84 months	0.03	(-0.03, 0.10)	0.33	0.04	(-0.02, 0.11)	0.17	0.01	(-0.06, 0.07)	0.79	0.00	(-0.06 , 0.07)	0.99

Model 1: adjusted for urban or rural residence and East or West of Belarus.

Model 2: as Model 1 additionally adjusted for mid-parental height.

Model 3: as Model 2 additionally adjusted for study trial arm, maternal smoking (never, ever or unknown) and older siblings (none, 1 or >1).

^1^Coef = Coefficient.

Controlling for urban/rural and East/West area of residence attenuated the association of maternal education with growth between birth and 3 months (model 1). Additionally controlling for mid-parental height (model 2), attenuated associations of birth length and length/height velocity with maternal education for all growth periods, amongst both girls and boys. However, further controlling for prolonged and exclusive breastfeeding, maternal smoking and the number of older siblings (model 3) made little difference to the results. The models were used to calculate the absolute height difference between the highest versus lowest categories of maternal education at age 7 years. For the unadjusted model, the absolute difference in height at 7 years was 1.92 cm (95% CI: 1.47, 2.36) taller among girls and 1.95 cm (95% CI: 1.53, 2.37) among boys; after controlling for urban/rural and East/West area of residence (model 1), this difference remained at 1.86 cm (95% CI: 1.42, 2.31) among girls and 1.89 cm (95% CI: 1.47, 2.31) among boys. Height differences at age 7 years attenuated by 40% after controlling for mid-parental height; the difference was 1.10 cm (95% CI: 0.69, 1.52) among girls and 1.16 cm (95% CI: 0.77, 1.55) among boys (model 2). On adjustment for prolonged and exclusive breastfeeding, maternal smoking and the number of older siblings (model 3), the difference remained [1.11 cm (95% CI: 0.69, 1.53) among girls and 1.08 cm (95% CI: 0.68, 1.47) among boys]; the fully adjusted differences in terms of WHO height-for-age standard deviations among girls was attenuated to 0.20 SD (95% CI: 0.13, 0.28) and to 0.20 SD (95% CI: 0.13, 0.28) among boys. However, even in the fully adjusted model, differences in growth between categories of maternal education persisted for birth length and growth in early childhood. Adjusting for mother’s height did not result in greater attenuation of the association of mother’s education with child’s growth trajectory compared to adjusting for father’s height (results not shown).

Findings for categories of paternal education (Additional file 1: Table S2) and highest household occupation (Additional file 1: Table S3) were similar to those for maternal education. Generally, among girls and boys, growth per category of paternal education or household occupation showed evidence of a difference in length/height growth rates in the unadjusted models at 0–3 and 12–34 months. The fully-adjusted models were used to calculate the absolute difference in height between the highest versus lowest categories of paternal education and non-manual versus manual household occupation at age 7 years; for girls, the height differences were 0.47 cm (95% CI: 0.06, 0.89) and 0.19 cm (95% CI: -0.10, 0.47), respectively; for boys, the differences were 0.15 cm (95% CI: -0.23, 0.54) and 0.42 cm (95% CI: 0.15, 0.69), respectively. The difference in terms of WHO height-for-age standard deviations among girls by paternal education was 0.09 SD (95% CI: 0.01, 0.16), and by highest household occupation was 0.03 SD (95% CI: -0.02, 0.09); the corresponding differences for boys were 0.03 SD (95% CI: -0.04, 0.10) and 0.08 SD (95% CI: 0.03, 0.13), respectively.

## Discussion

Amongst over 12,000 healthy, term infants from the Republic of Belarus, a middle-income former Soviet country, children born to the most educated parents were longer at birth and grew faster in stature in early infancy and early childhood than those born to the least educated parents, with some widening of socioeconomic differences with age. Findings were similar on comparing children from households with non-manual occupations to those from households with manual occupations. After adjusting for confounders, controlling for mid-parental height moderately attenuated these associations. However, controlling for prolonged and exclusive breastfeeding, maternal smoking and older siblings did not substantially alter the associations between maternal education and growth.

The parents of the PROBIT children were born under the communist system, in which education (including universities) was universal and free. Our findings of a positive relationship between parental education or highest household occupation and length/height growth are broadly consistent with some [18, 23, 26–28, 54] but not all [24] studies in various settings. Previous studies have used different statistical methods [26–28, 54] from ours and/or alternative measures of socioeconomic position [26, 27]. Two recent papers have modelled growth trajectories using the same methods as the present study. One contemporary British cohort (Avon Longitudinal Study of Parents and Children, ALSPAC) found that birth length was shortest amongst children of the least educated women, with a small widening of differences up to 10 years of age [23]. In Southern Brazil, amongst infants born and followed for 4 years, maternal education was similarly positively associated with birth length and also positively associated with growth rates [18]. Although these studies were set in countries with different economies (the U.K. is a high- and Brazil a middle-income country), both observed that children from the most educated mothers were longer at birth-as we did in Belarus. In Brazil, children of the highest-educated mothers grew faster than the children of the lowest-educated mothers, with a widening of differences with age: from 0.2 SD of birth length to 0.7 SD of height at age 4 years. In Belarus, the difference between extreme categories of maternal education was approximately 0.2 SD at birth and 0.4 SD at age 4 years (in WHO SD). Our findings in Belarus are thus similar to those from Brazil, but a greater widening of socioeconomic differences was reported at age 4 years in Brazil.

Few studies have examined the effect of possible mediators/confounders on the association of socioeconomic position with birth length and rate of growth in children. We observed an average 40% further reduction in socioeconomic height differences after adjustment for mid-parental height, beyond the reduction due to adjustment for confounders. Using similar methods to our study and with comparable findings, the Brazilian cohort also found that adjusting for maternal height (rather than mid-parental height) attenuated socioeconomic differences in birth length and (as in our study) growth rates in early infancy and early childhood [18]. Another study from the ALSPAC cohort found the socioeconomic difference in offspring height change was attenuated after controlling for mid-parental height [54], whilst others have reported that adding maternal and paternal height with other covariables resulted in greater socioeconomic differences [24].

The mechanisms leading to socioeconomic differences in growth require further research. The literature suggests that maternal education may influence offspring length/height trajectories through the educated mother’s environment, income and behaviour, including the ability to access and utilise health care information and services during pregnancy and postnatally [55]. As described above, we found mid-parental height explained some of the socioeconomic differences in length/height trajectories. The final attained height of each parent reflects a complex combination of his or her accumulated inherited genetic material and the fetal, infant and childhood environment in which they grew up [14]. In turn, these factors may also play a part in the offspring’s growth trajectory with any genetic height potential only being reached if the environment allows. Length/height differences widen from birth to 3 months, but on adjustment for mid-parental height, the differences were reduced substantially, suggesting that parental height explains some of the differences in growth rates during this period. Height differences also widen notably between 1–3 years; while mid-parental height explains some of the difference, other factors related to socioeconomic position, such as family diet and exposure to illness/infection, may influence offspring health and hence height growth during this period. Our findings suggest that public health interventions to improve socioeconomic conditions in early childhood, such as promoting healthy diets, educating parents about improving uptake of immunisations, preventing illness/infection and seeking early medical care in case of illness might have a beneficial impact on later height and health.

We are continuing to assess growth in this cohort as the children progress through puberty and into adulthood to determine the most important periods of growth associated with adult attained height and mortality. Further studies could also determine important modifiable factors, which may suggest interventions applied at suitable ages, aimed at reducing socioeconomic differences in height. Our findings may not be generalizable to other settings, for example, our height differences were fairly modest compared to those seen in the Brazilian cohort [18], mentioned above. Further investigation of longitudinal cohorts in other middle-income populations with similar or different economic histories would add to our understanding of socioeconomic differences in childhood growth.

The main strength of the study was our large sample size with many measures of length/height over a long period; a growth modelling strategy which utilized all of the available data; analysis that was not restricted to individuals with complete data at all time points or data measured at exactly the same ages for all individuals; and allowed for the change in size and variation of length/height with age. One limitation of our study is that measurements in infancy and childhood were based on routine child health records (only measurements at the 6.5-year follow-up were standardised and audited), so associations may have been attenuated by measurement error. We are unable to assess the reliability of the routinely-collected length/height measurements in Belarus. We combined length and height measurements, even though they are measured differently. Recumbent length can be very difficult to measure accurately and tends to be longer than standing height; the difference between these two measurement methods in children aged 2–3 years can be as much as 0.47 cm [56]. We were unable to control for method of length/height measurement (whether recumbent or standing) as this was not recorded; therefore associations may be attenuated by this measurement error. The difference between the 16,861 children with at least two measures of length/height compared to those 12,463 with complete data, showed that those with missing data were more likely to have less educated mothers and more likely to be slightly shorter at birth. These differences should not, however, affect the associations we observed between socioeconomic status and growth trajectories. We found adjustment for breastfeeding (by study trial arm), maternal smoking and older siblings did not alter the association between maternal education and offspring growth substantively. This suggests that either maternal education does not mediate its effect on offspring growth through these factors, or that by controlling for a mediator we have induced another association (e.g. induced confounding) which masks the effect of the mediator.

## Conclusion

In summary, despite low reported levels of income inequality [30], socioeconomic differences in length/height growth amongst children from Belarus were present at birth and widened through early infancy and early childhood. These differences were partly explained by mid-parental height, suggesting that factors influencing the parents’ growth during their own childhood have a bearing on these socioeconomic differences. Our findings suggest that public health interventions aimed at improving socioeconomic conditions in early childhood might help reduce socioeconomic differences in length/height in this setting.

## Electronic supplementary material

Additional file 1: Table S1: Observed mean length/height measurements (cm) and mean World Health Organization (WHO) child growth standards z-score by sex, age, and maternal education. **Table S2.** Crude & multiple analysis of mean growth rate per category of paternal education, N = 12,203 **Table S3.** Crude & multiple analysis of growth rate in manual versus non-manual highest household occupation, N = 11,761. Supplementary information. (DOCX 43 KB)
