# Supplementary material for: Socioeconomic differences in childhood length/height trajectories in a middle-income country: a cohort study
Source: BMC Public Health. 2014 Sep 8;14:932. doi: 10.1186/1471-2458-14-932 (PMC4181044; doi:10.1186/1471-2458-14-932)
Supplement: Supplementary file 1 — Additional file 1: Table S1: Observed mean length/height measurements (cm) and mean World Health Organization (WHO) child growth standards z-score by sex, age, and maternal education. Table S2. Crude & multiple analysis of mean growth rate per category of paternal education, N = 12,203 Table S3. Crude & multiple analysis of growth rate in manual versus non-manual highest household occupation, N = 11,761. Supplementary information. (DOCX 43 KB) [file 12889_2013_7090_MOESM1_ESM.docx]

**Supplementary Information**

**Table S1 Observed mean length/height measurements (cm) and mean World Health Organization (WHO) child growth standards z-score by sex, age, and maternal education.**

|  |  | **Initial,** **incomplete or common secondary** | | |  | **Advance secondary or partial university** | | |  | **Completed university** | | |
| --- | --- | --- | --- | --- | --- | --- | --- | --- | --- | --- | --- | --- |
| **Age range in years** |  | **N** | **Length**  **/height, cm**  **(SD)** | **WHO**  **z-score**  **(SD)** |  | **N** | **Length**  **/height, cm**  **(SD)** | **WHO**  **z-score**  **(SD)** |  | **N** | **Length**  **/height, cm**  **(SD)** | **WHO**  **z-score**  **(SD)** |
| **Girls** |  |  |  |  |  |  |  |  |  |  |  |  |
| **Birth*** |  | 2,075 | 51.45 (2.05) | 1.23 (1.09) |  | 3,099 | 51.73 (2.05) | 1.38 (1.09) |  | 836 | 51.90 (2.01) | 1.48 (1.08) |
| **0.2-0.25** |  | 593 | 59.63 (2.30) | 0.02 (1.07) |  | 847 | 59.95 (2.25) | 0.18 (1.04) |  | 238 | 60.00 (2.27) | 0.18 (1.05) |
| **0.4-0.5** |  | 674 | 65.81 (2.58) | 0.12 (1.14) |  | 907 | 66.25 (2.45) | 0.31 (1.08) |  | 229 | 66.41 (2.58) | 0.34 (0.96) |
| **0.7-0.75** |  | 654 | 70.56 (2.51) | 0.23 (1.04) |  | 933 | 71.17 (2.44) | 0.49 (1.01) |  | 233 | 71.26 (2.17) | 0.51 (0.84) |
| **0.8-1** |  | 831 | 74.46 (2.95) | 0.39 (1.08) |  | 1,177 | 74.89 (2.78) | 0.55 (1.00) |  | 311 | 75.23 (2.95) | 0.65 (1.03) |
| **1.8-2** |  | 381 | 84.79 (4.27) | -0.26 (1.35) |  | 543 | 85.66 (4.23) | 0.00 (1.31) |  | 146 | 86.32 (4.73) | 0.13 (1.23) |
| **2.8-3** |  | 305 | 93.23 (4.83) | -0.30 (1.24) |  | 478 | 94.28 (4.62) | -0.01 (1.22) |  | 120 | 94.57 (4.46) | 0.07 (1.19) |
| **3.8-4** |  | 226 | 101.34 (5.59) | -0.18 (1.32) |  | 395 | 101.95 (4.97) | -0.04 (1.17) |  | 111 | 102.52 (5.06) | 0.10 (1.21) |
| **4.8-5** |  | 280 | 108.57 (5.24) | -0.08 (1.10) |  | 396 | 109.01 (5.09) | 0.03 (1.08) |  | 100 | 109.73 (5.72) | 0.18 (1.22) |
| **5.8-6** |  | 239 | 115.17 (5.40) | 0.11 (1.06) |  | 405 | 115.85 (4.84) | 0.24 (0.95) |  | 108 | 117.21 (5.28) | 0.50 (1.04) |
| **6.4-6.5** |  | 488 | 118.32 (5.05) | 0.11 (0.95) |  | 800 | 119.55 (4.87) | 0.34 (0.91) |  | 229 | 120.48 (4.64) | 0.52 (0.88) |
| **6.8-7** |  | 117 | 121.44 (5.11) | 0.22 (0.94) |  | 160 | 122.38 (5.34) | 0.41 (0.99) |  | 49 | 123.48 (5.00) | 0.61 (0.93) |
| **Boys** |  |  |  |  |  |  |  |  |  |  |  |  |
| **Birth*** |  | 2,252 | 52.19 (2.15) | 1.20 (1.11) |  | 3,321 | 52.41 (2.19) | 1.31 (1.12) |  | 880 | 52.48 (2.14) | 1.36 (1.10) |
| **0.2-0.25** |  | 640 | 60.68 (2.50) | -0.26 (1.21) |  | 913 | 61.14 (2.47) | -0.03 (1.18) |  | 226 | 61.24 (2.30) | 0.02 (1.09) |
| **0.4-0.5** |  | 701 | 67.13 (2.65) | -0.13 (1.24) |  | 1,005 | 67.66 (2.56) | 0.12 (1.19) |  | 257 | 67.90 (2.44) | 0.23 (1.13) |
| **0.7-0.75** |  | 684 | 71.81 (2.63) | 0.01 (1.14) |  | 953 | 72.29 (2.39) | 0.21 (1.06) |  | 260 | 72.47 (2.46) | 0.30 (1.10) |
| **0.8-1** |  | 884 | 75.56 (3.16) | 0.13 (1.17) |  | 1,243 | 75.89 (3.04) | 0.30 (1.17) |  | 339 | 76.38 (2.85) | 0.53 (1.18) |
| **1.8-2** |  | 383 | 85.76 (4.24) | -0.45 (1.37) |  | 628 | 86.38 (4.12) | -0.23 (1.35) |  | 162 | 87.18 (3.96) | 0.05 (1.34) |
| **2.8-3** |  | 320 | 94.19 (4.37) | -0.33 (1.20) |  | 520 | 94.87 (4.61) | -0.14 (1.27) |  | 126 | 95.37 (4.02) | 0.01 (1.11) |
| **3.8-4** |  | 255 | 101.43 (4.95) | -0.32 (1.18) |  | 427 | 102.71 (4.87) | 0.01 (1.18) |  | 91 | 103.51 (5.04) | 0.22 (1.20) |
| **4.8-5** |  | 249 | 108.97 (5.05) | -0.09 (1.11) |  | 418 | 109.93 (5.34) | 0.11 (1.16) |  | 110 | 110.72 (5.10) | 0.28 (1.11) |
| **5.8-6** |  | 261 | 115.14 (4.94) | -0.05 (0.95) |  | 437 | 116.69 (4.85) | 0.26 (0.99) |  | 106 | 117.65 (5.31) | 0.46 (1.10) |
| **6.4-6.5** |  | 522 | 119.13 (4.74) | 0.10 (0.93) |  | 818 | 119.82 (4.77) | 0.24 (0.94) |  | 206 | 120.34 (4.50) | 0.34 (0.88) |
| **6.8-7** |  | 145 | 121.54 (4.76) | 0.08 (0.90) |  | 185 | 122.68 (5.32) | 0.29 (1.01) |  | 58 | 124.58 (5.34) | 0.64 (1.00) |

Note: *Inclusion criteria specified that the infants at birth were full-term (≥37 weeks gestation) healthy singletons, weighed at least 2,500 g at birth and had a 5-minute Apgar score ≥5. Hence z-score at birth values are higher than WHO reference population.

SD=Standard deviation

**Table S2 Crude & multiple analysis of mean growth rate per category of paternal education, N=12,203**

|  | **Per category of father’s education change in length/height rate** | | | | | | | | | | | | | | |
| --- | --- | --- | --- | --- | --- | --- | --- | --- | --- | --- | --- | --- | --- | --- | --- |
|  | **Unadjusted model** | | |  | **Model 1** | | |  | **Model 2** | | |  | **Model 3** | | |
|  | **^1^Coef** | **95%CI** | **P for trend** |  | **Coef** | **95%CI** | **P for trend** |  | **Coef** | **95%CI** | **P for trend** |  | **Coef** | **95%CI** | **P for trend** |
| **Girls N=5,884** |  |  |  |  |  |  |  |  |  |  |  |  |  |  |  |
| Birth length, cm | 0.14 | (0.06, 0.21) | <0.001 |  | 0.16 | (0.09, 0.24) | <0.001 |  | 0.13 | (0.05, 0.20) | 0.001 |  | 0.11 | (0.03, 0.18) | 0.004 |
| Growth, cm/year:  0-3 months | 0.29 | (-0.05, 0.63) | 0.09 |  | 0.07 | (-0.26, 0.41) | 0.67 |  | -0.03 | (-0.37, 0.30) | 0.84 |  | 0.01 | (-0.33, 0.34) | 0.98 |
| >3-12 months | -0.05 | (-0.18, 0.09) | 0.49 |  | -0.06 | (-0.19, 0.08) | 0.42 |  | -0.10 | (-0.24, 0.03) | 0.14 |  | -0.10 | (-0.24, 0.03) | 0.14 |
| >12-34 months | 0.24 | (0.12, 0.37) | <0.001 |  | 0.20 | (0.08, 0.33) | 0.002 |  | 0.16 | (0.03, 0.29) | 0.01 |  | 0.19 | (0.06, 0.31) | 0.004 |
| >34-84 months | -0.01 | (-0.07, 0.06) | 0.81 |  | 0.01 | (-0.06, 0.07) | 0.87 |  | -0.03 | (-0.09, 0.04) | 0.40 |  | -0.03 | (-0.10, 0.03) | 0.33 |
|  |  |  |  |  |  |  |  |  |  |  |  |  |  |  |  |
| **Boys N=6,319** |  |  |  |  |  |  |  |  |  |  |  |  |  |  |  |
| Birth length, cm | 0.06 | (-0.02, 0.14) | 0.14 |  | 0.10 | (0.02, 0.17) | 0.01 |  | 0.05 | (-0.03, 0.13) | 0.20 |  | 0.03 | (-0.04, 0.11) | 0.37 |
| Growth, cm/year:  0-3 months | 0.91 | (0.57, 1.25) | <0.001 |  | 0.60 | (0.27, 0.93) | <0.001 |  | 0.49 | (0.15, 0.82) | 0.004 |  | 0.52 | (0.19, 0.85) | 0.002 |
| >3-12 months | -0.02 | (-0.16, 0.11) | 0.72 |  | -0.03 | (-0.16, 0.11) | 0.70 |  | -0.06 | (-0.19, 0.08) | 0.42 |  | -0.07 | (-0.21, 0.07) | 0.31 |
| >12-34 months | 0.25 | (0.13, 0.37) | <0.001 |  | 0.23 | (0.11, 0.35) | <0.001 |  | 0.18 | (0.06, 0.30) | 0.003 |  | 0.19 | (0.07, 0.31) | 0.002 |
| >34-84 months | -0.06 | (-0.13, 0) | 0.04 |  | -0.05 | (-0.12, 0.01) | 0.09 |  | -0.09 | (-0.15, -0.02) | 0.007 |  | -0.09 | (-0.15, -0.03) | 0.005 |

Model 1: adjusted for urban or rural residence and East or West of Belarus

Model 2: as Model 1 additionally adjusted for mid-parental height

Model 3: as Model 2 additionally adjusted for study trial arm, maternal smoking (never, ever or unknown) and older siblings (none, 1 or >1)

^1^ Coef=Coefficient

**Table S3 Crude & multiple analysis of growth rate in manual versus non-manual highest household occupation, N=11,761**

|  | **Manual to non-manual change in length/height rate** | | | | | | | | | | | | | | |
| --- | --- | --- | --- | --- | --- | --- | --- | --- | --- | --- | --- | --- | --- | --- | --- |
|  | **Unadjusted model** | | |  | **Model 1** | | |  | **Model 2** | | |  | **Model 3** | | |
|  | **^1^Coef** | **95%CI** | **P for trend** |  | **Coef** | **95%CI** | **P for trend** |  | **Coef** | **95%CI** | **P for trend** |  | **Coef** | **95%CI** | **P for trend** |
| **Girls N=5,686** |  |  |  |  |  |  |  |  |  |  |  |  |  |  |  |
| Birth length, cm | 0.23 | (0.13, 0.34) | <0.001 |  | 0.25 | (0.15, 0.36) | <0.001 |  | 0.20 | (0.10, 0.31) | <0.001 |  | 0.20 | (0.09, 0.30) | <0.001 |
| Growth, cm/year:  0-3 months | 0.36 | (-0.11, 0.84) | 0.13 |  | 0.21 | (-0.26, 0.68) | 0.38 |  | 0.05 | (-0.42, 0.52) | 0.83 |  | 0.03 | (-0.44, 0.50) | 0.89 |
| >3-12 months | 0.05 | (-0.14, 0.24) | 0.62 |  | 0.05 | (-0.14, 0.24) | 0.62 |  | -0.02 | (-0.21, 0.17) | 0.85 |  | -0.03 | (-0.22, 0.16) | 0.77 |
| >12-34 months | 0.23 | (0.05, 0.41) | 0.01 |  | 0.19 | (0.01, 0.37) | 0.03 |  | 0.12 | (-0.05, 0.30) | 0.17 |  | 0.14 | (-0.04, 0.32) | 0.13 |
| >34-84 months | -0.01 | (-0.10, 0.09) | 0.91 |  | 0.00 | (-0.09, 0.09) | 0.99 |  | -0.05 | (-0.14, 0.04) | 0.28 |  | -0.06 | (-0.15, 0.03) | 0.21 |
|  |  |  |  |  |  |  |  |  |  |  |  |  |  |  |  |
| **Boys N=6,075** |  |  |  |  |  |  |  |  |  |  |  |  |  |  |  |
| Birth length, cm | 0.15 | (0.04, 0.25) | 0.008 |  | 0.17 | (0.06, 0.28) | 0.002 |  | 0.09 | (-0.02, 0.2) | 0.10 |  | 0.10 | (-0.01, 0.20) | 0.08 |
| Growth, cm/year:  0-3 months | 0.73 | (0.26, 1.21) | 0.003 |  | 0.52 | (0.06, 0.99) | 0.03 |  | 0.32 | (-0.15, 0.79) | 0.18 |  | 0.29 | (-0.18, 0.76) | 0.22 |
| >3-12 months | 0.12 | (-0.07, 0.31) | 0.23 |  | 0.12 | (-0.07, 0.31) | 0.22 |  | 0.07 | (-0.12, 0.26) | 0.49 |  | 0.03 | (-0.17, 0.22) | 0.77 |
| >12-34 months | 0.25 | (0.09, 0.42) | 0.003 |  | 0.24 | (0.07, 0.41) | 0.005 |  | 0.17 | (0, 0.33) | 0.06 |  | 0.16 | (-0.01, 0.33) | 0.07 |
| >34-84 months | 0.05 | (-0.04, 0.14) | 0.30 |  | 0.05 | (-0.04, 0.14) | 0.27 |  | -0.01 | (-0.10, 0.08) | 0.88 |  | -0.01 | (-0.10, 0.08) | 0.78 |

Model 1: adjusted for urban or rural residence and East or West of Belarus

Model 2: as Model 1 additionally adjusted for mid-parental height

Model 3: as Model 2 additionally adjusted for study trial arm, maternal smoking (never, ever or unknown) and older siblings (none, 1 or >1)

^1^ Coef=Coefficient

**Supplementary information**

The models were used to calculate the absolute height difference between the highest versus lowest categories of maternal education at age 7 years using the *lincom* command in STATA and the following formula:

[coefficient birth length]*2 + 0.25*[coefficient 0-3 months]*2 + 0.75*[coefficient 3-12 months]*2 + 1.83*[coefficient 12-34 months] *2 + 4.167*[coefficient 34-84 months]*2

**Note:**

1. The coefficients represent the interaction between maternal education and the time period.
2. Maternal education is recorded as one of three categories. In this model, the socioeconomic indicator is fitted as a continuous variable, so we assume a jump of two units from the lowest to the highest category of maternal education.
